# Supplementary material for: The Short-Term Opening of Cyclosporin A-Independent Palmitate/Sr2+-Induced Pore Can Underlie Ion Efflux in the Oscillatory Mode of Functioning of Rat Liver Mitochondria
Source: Membranes (Basel). 2022 Jun 28;12(7):667. doi: 10.3390/membranes12070667 (PMC9319229; doi:10.3390/membranes12070667)
Supplement: Supplementary file 1 [file membranes-12-00667-s001.zip › membranes-1786488-supplementary.pdf]

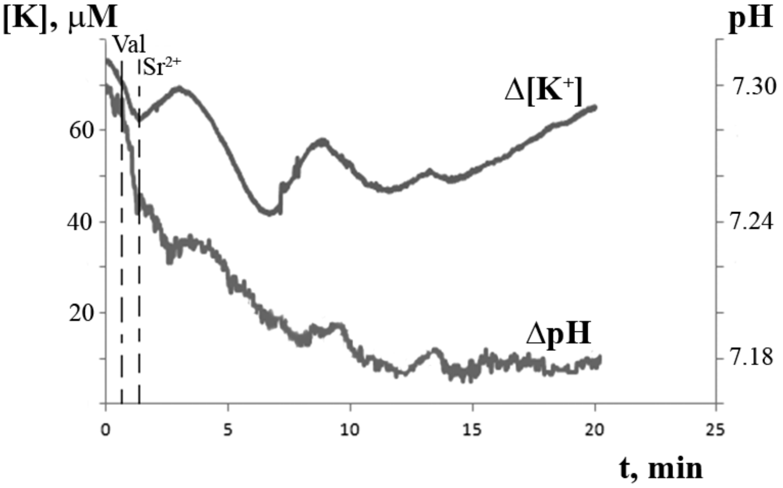


**Figure S1.** Simultaneous recording of $Sr^{2+}$/valinomycin-induced oscillations in $K^+$ fluxes and external pH in the suspension of rat liver mitochondria. The medium contained 20 mM sucrose, 1 mM KCl, 1 μM CsA, 1 μM rotenone, 5 mM succinic acid, 12.5 mM Tris (pH 7.3). Additions: 2 ng valinomycin and 45 nmol $SrCl_2$/mg of protein. Changes in the concentrations of $K^+$ and $H^+$ were recorded simultaneously in a 1-ml temperature-controlled cell with constant stirring at 26 C using ion-selective electrodes. The typical traces are presented (*n* = 5).

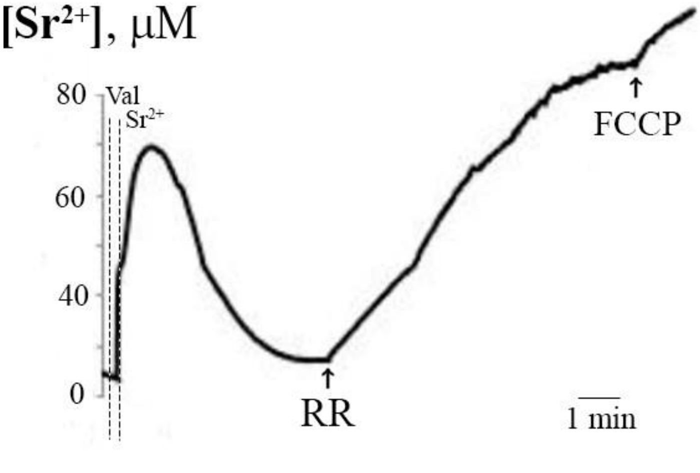


**Figure S2.** Ruthenium red (RR, 1 μM) prevents the extension of the $Sr^{2+}$/valinomycin-induced cyclic changes in strontium ion fluxes in rat liver mitochondria. The medium and experimental conditions were the same as in Figure S1. Additions: 2 ng valinomycin and 45 nmol $SrCl_2$/mg of protein. The concentrations of $Sr^{2+}$ ions in the incubation medium were determined with an ion-selective electrode and an electrometrical system Record 4 (Russia). The typical traces are presented (*n* = 5).

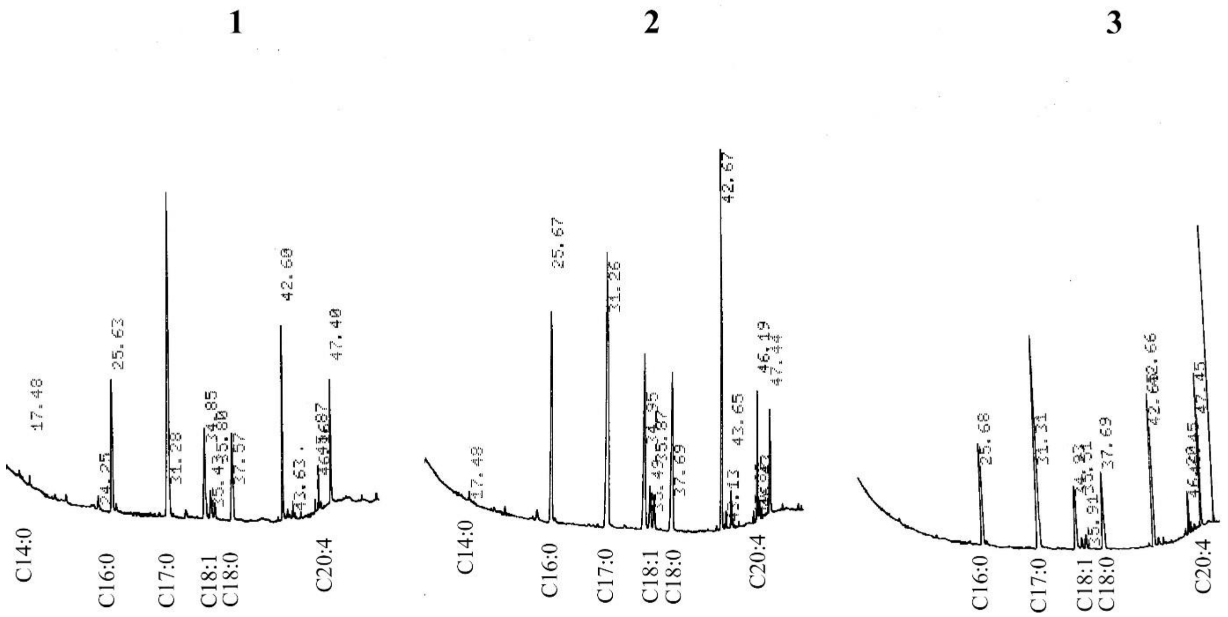


**Figure S3.** Gas-liquid chromatography profile of FFA methyl esters obtained from rat liver mitochondria before (control, **1**) and after the onset of $Sr^{2+}$/valinomycin-induced ion oscillations in the absence (0.1% DMSO) (**2**) or presence of 25 μM aristolochic acid (ArA), a phospholipase $A_2$ inhibitor (**3**). Chromatograms were obtained on a glass open tubular column packed with Porapak Q (2mm i.d. x 1m) installed in a Pye-Unicam Model 304 gas chromatograph as described in the Material and Methods section. The typical chromatograms are presented (*n* = 4).

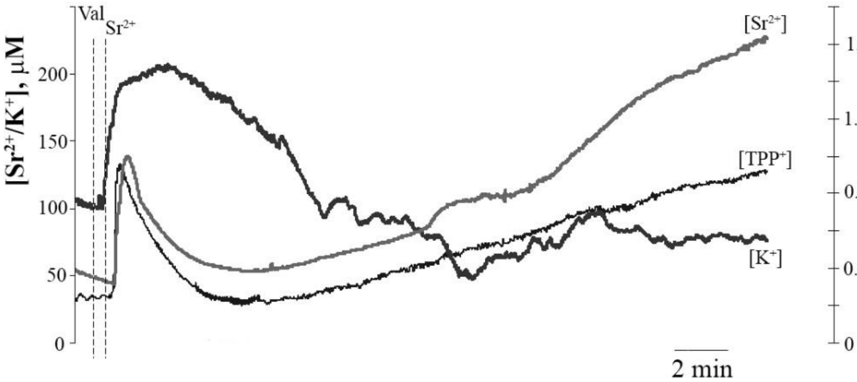


**Figure S4.** Blocking effect of $AACOCF_3$ (15 μM), a $Ca^{2+}$-dependent phospholipase $A_2$ inhibitor, on $Sr^{2+}$/valinomycin-induced cyclic changes in the fluxes of $Sr^{2+}$, $K^+$, and $TPP^+$ in rat liver mitochondria. The medium and conditions were the same as in Figure S1. Changes in the concentrations of $Sr^{2+}$, $K^+$, and $TPP^+$ ions were recorded simultaneously in a 1-ml temperature-controlled cell using an original multichannel electrometrical system Record 4 (Russia) and ion-sensitive electrodes. Additions: 2 ng valinomycin and 45 nmol $SrCl_2$/mg of protein. The $PLA_2$ inhibitor were added to the mitochondria 1 min before the addition of $Sr^{2+}$. The typical traces are presented (*n* = 5).

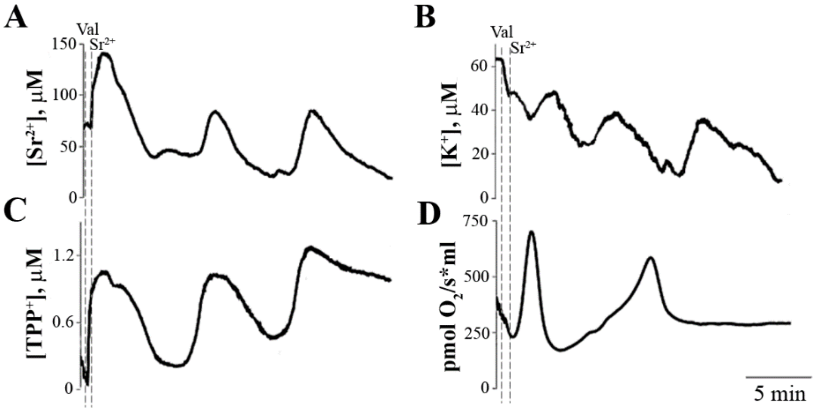


**Figure S5.** The $Ca^{2+}$-independent phospholipase $A_2$ inhibitor PACOCF3 (20 µM) has no effect on $Sr^{2+}$/valinomycin-induced cyclic changes in the fluxes of $Sr^{2+}$ (A), $K^+$ (B), $TPP^+$ (C), and the respiration rate (D) of rat liver mitochondria. The medium and conditions were the same as in Figure S1. Additions: 2 ng valinomycin and 45 nmol $SrCl_2$/mg of protein. The $PLA_2$ inhibitor was added to the mitochondria 1 min before the addition of $Sr^{2+}$. The typical traces are presented (*n* = 5).

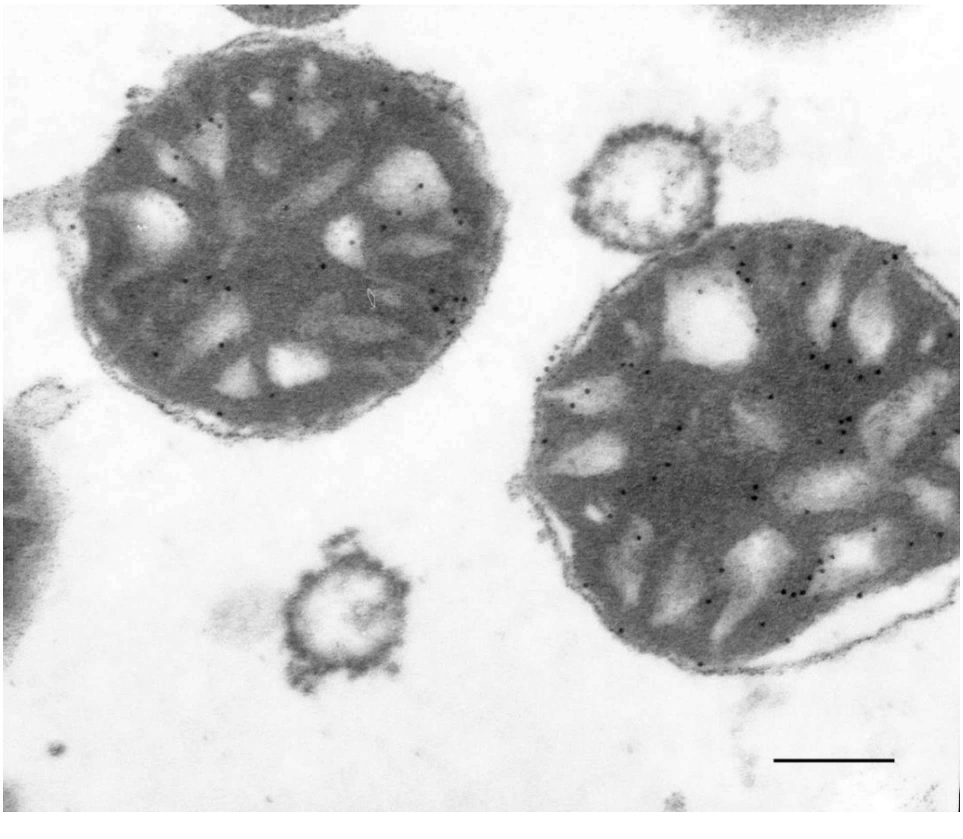

**(a)**

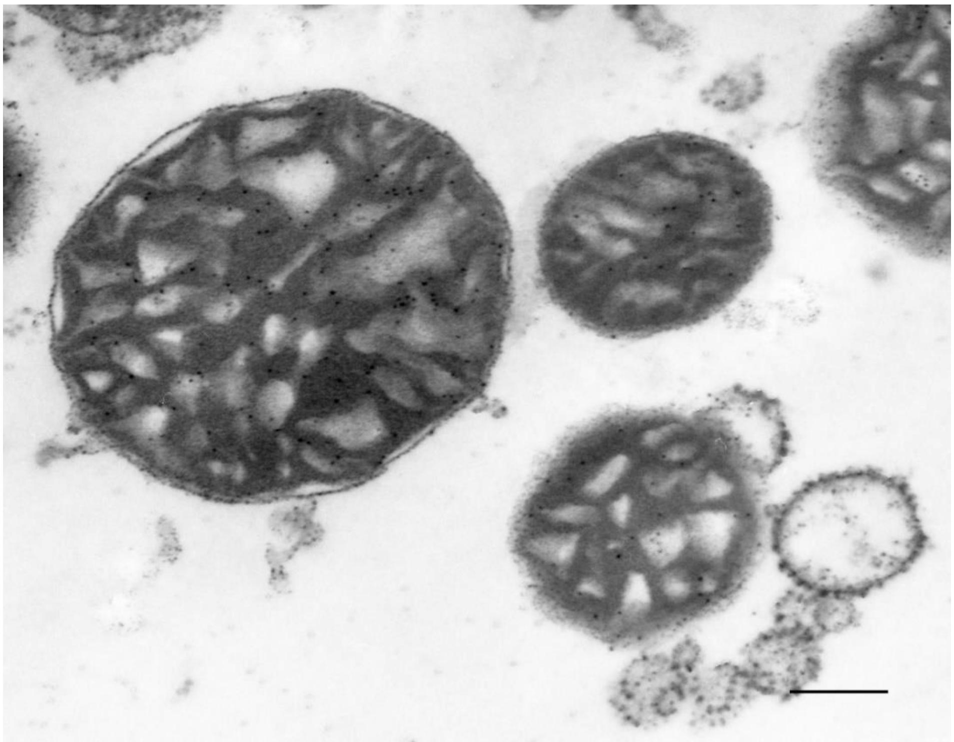

**(b)**

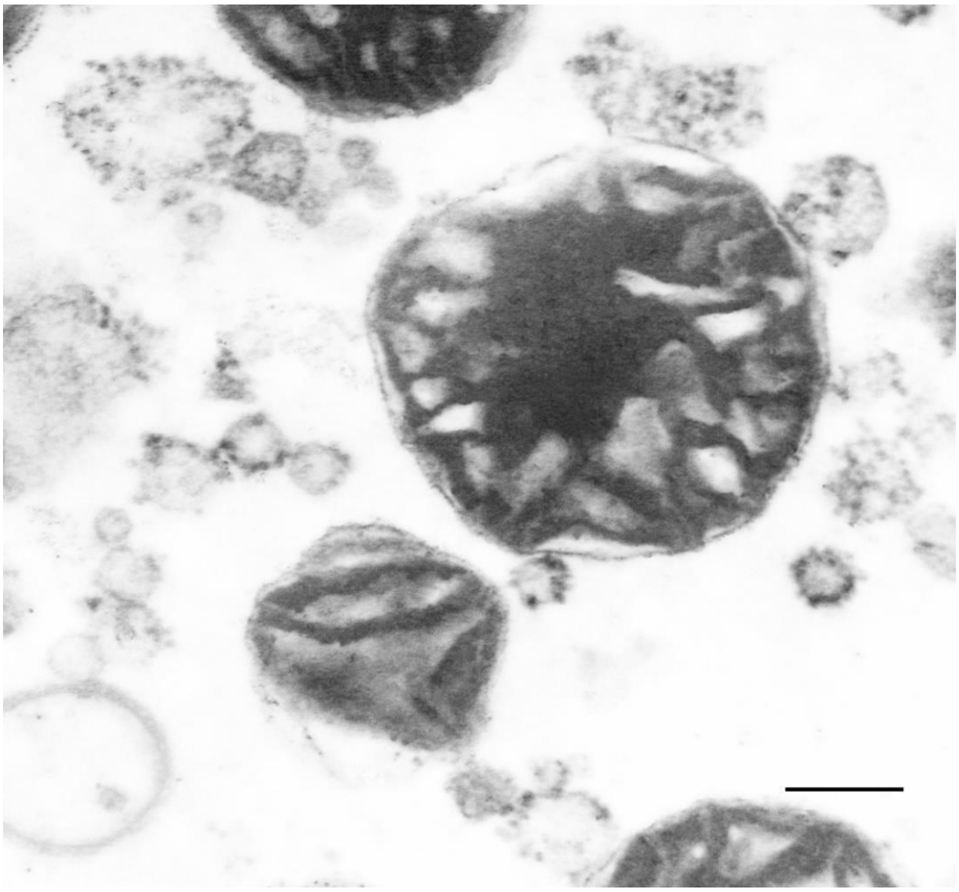

**(c)**

**Figure S6.** Typical immunoelectron micrographs of isolated rat liver mitochondria treated with antibodies against the group IV cytosolic phospholipase A2 (**a**, **b**) or 0.1 M PBS buffer (**c**) in the

presence of 10-nm colloidal gold labeled antibodies: (**a, b**) Mitochondria were incubated with specific antibodies against the group IV $Ca^{2+}$($Sr^{2+}$)-dependent cPLA2 (Santa Cruz Biotechnology, USA) and secondary antibodies labeled with colloidal gold with a particle size of 10 nm (Sigma-Aldrich, St. Louis, MO, USA). Black granules are the binding sites for the antibodies and the protein; (**c**) Control; mitochondria were incubated in 0.1 M PBS buffer instead of anti-cPLA2 antibody. Scale bar, 0.25 μm.
